# ﻿Two new species of terrestrial microsnails of the genus *Hypselostoma* W.H. Benson, 1856 (Gastropoda, Eupulmonata, Hypselostomatidae) from northeastern Thailand

**DOI:** 10.3897/zookeys.1265.160661

**Published:** 2025-12-23

**Authors:** Kitti Tanmuangpak, Benchawan Nahok, Utain Chanlabut, Chanidaporn Tumpeesuwan, Sakboworn Tumpeesuwan

**Affiliations:** 1 Program of Biology, Department of Science, Faculty of Science and Technology, Loei Rajabhat University, Mueang District, Loei 42000, Thailand Loei Rajabhat University Loei Thailand; 2 Program of General Science, Faculty of Education and Human Development, Chaiyaphum Rajabhat University, Mueang District, Chaiyaphum 36000, Thailand Chaiyaphum Rajabhat University Chaiyaphum Thailand; 3 Department of Biology, Faculty of Science, Mahasarakham University, Kantharawichai District, Maha Sarakham 44150, Thailand Mahasarakham University Maha Sarakham Thailand

**Keywords:** Apertural barriers, double-keeled last whorl, genital system, limestone hills, radula

## Abstract

Two new species of hypselostomatid land microsnails, *Hypselostoma pongrati* Tanmuangpak & S. Tumpeesuwan, **sp. nov.** from Nong Bua Lamphu Province and *H. sichomphuense* Tanmuangpak & S. Tumpeesuwan, **sp. nov.** from Khon Kaen Province, are described based on the morphology of their shell, radula, and genital system. Both new species are very similar in shell shape to *H. phupaman* from Chaiyaphum Province, possessing a double-keeled last whorl. However, *H. pongrati* Tanmuangpak & S. Tumpeesuwan, **sp. nov.** is distinguished by the absence of apertural barriers, whereas *H. sichomphuense* Tanmuangpak & S. Tumpeesuwan, **sp. nov.** possesses three apertural barriers, and *H. phupaman* possesses six or seven. The three species were found on isolated limestone hills in the Chaiyaphum-Khon Kaen-Nong Bua Lamphu-Loei area.

## ﻿Introduction

**Figure 1.**
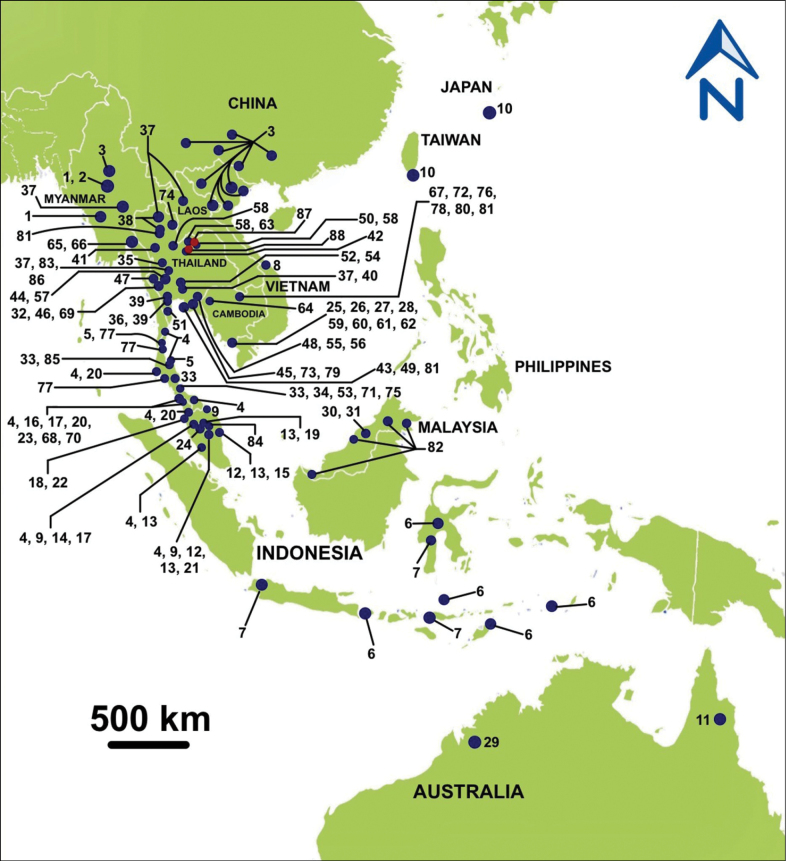
Map of Southeast Asia and the surrounding area showing the type localities of the known *Hypselostoma* spp. The numbers correspond to the species numbers listed in Table 1.

The land pulmonate microsnails of the genus *Hypselostoma* W.H. Benson, 1856, were classified into the superfamily Pupilloidea W. Turton, 1831 and family Hypselostomatidae Zilch, 1959 (Gojšina et al. 2025), based on their shell morphology, especially apertural barriers (van Benthem Jutting 1950; Schileyko 1998; Panha and Burch 2008; Sutcharit et al. 2023). This genus has high-spired, conical shells with a free trumpet-shaped last whorl. Panha and Burch (2008) used the separation of the angular and parietal lamellae within the aperture as the key character to distinguish *Gyliotrachela* Tomlin, 1930 from *Hypselostoma*. Recently, Gojšina et al. (2025) treated *Gyliotrachela* and *Antroapiculus* Panha & J.B. Burch, 2002 as junior synonyms of *Hypselostoma*, because there are several instances of species from both genera having very similar, if not identical, shell morphologies, while the presence of parietal angular lamellae alone would not justify the separation of the two genera. In their taxonomic revision, Gojšina et al. (2025) concluded that the updated number of species of *Hypselostoma* is now 95 species (85 verified species + 10 unverified species from the Philippines), and that this genus is widely distributed in Southeast Asia and north and west Australia, Japan, and China (van Benthem Jutting 1950, 1962; Solem 1981; Schileyko 1998; Panha et al. 2004; Panha and Burch 2008; Tongkerd et al. 2013; Gojšina et al. 2025) (Table 1, Fig. 1).

**Table 1.** Nominal species and subspecies of *Hypselostoma* and their reported distribution (excluding the uncertain records from the Philippines).

S/N	Species name	Localities	References
1	*H. tubiferum* (W.H. Benson, 1856)	Thyet-Mio, Irawadi Burmanici, Myanmar	3
2	*H. bensonianum* W.T. Blanford, 1863	Mandalay Division, Myanmar	1, 3, 4, 18
3	*H. crossei* Morlet, 1886	Lang-son, Tonkin, Vietnam; Tianxingqiao Town, Zhenning Bouyeizu Miaozu Zizhixian, Guizhou, China; Nawit Village, Viengxay District, Houaphane, Laos	3, 6, 25
4	*H. hungerfordianum* Möllendorff, 1891	Bukit Baling, Kedah; Gunong Pondok, Perak; Batu Caves, Selangor; Bukit Chintamani and Gua Bama, Pahang, Malaysia; Kuankalong Limestone Hill, Satun; Samui Islands, Thailand	3, 7, 8, 29
5	*H. transitans* Möllendorff, 1894	Samui Islands, Surat Thani and Chumphon, Thailand	3, 25, 26, 33
6	*H. everetti* E.A. Smith, 1896	Timor Island, Kalao Island, Sulawesi, Java, Bali, Lesser Sunda Islands and Tanimbar Islands, Indonesia	3, 21, 33
7	*H. fruhstorferi* Möllendorff, 1897	West Java Province, Djampang, Java, and Makalé to Kalossi, Sulawesi, South Celebes, Indonesia	3, 30
8	*H. annamiticum* Möllendorff, 1900	Phuc-Son, Touranne, Annam, Vietnam	3
9	*H. kelantanense* (Sykes, 1902)	Kelantan and Kota Tongkat, Pahang, and Kramat Pulai, Perak, Malaysia	3
10	*H. insularum* Pilsbry, 1908	Yonakunijima, Ryuyku, Japan; Kenting Tropical Botanical Garden, Hengchun, Pingtung, Taiwan	3
11	*H. australe australe* Odhner, 1917	Chillagoe Caves, Queensland, Australia	3, 22
12	*H. terae* Tomlin, 1939	Bukit Chintamani, Pahang, and Bukit Charas, Pahang, and Bukit Takun, Kanching, Selangor, Malaysia	3
13	*H. depressispira* (van Benthem Jutting, 1949)	Bukit Chintamani; Gua Bama, Pahang, Bukit Panching, Pahang Malaysia	3, 7, 29
14	*H. piconis* van Benthem Jutting, 1949	Sungei Siput, Perak, Malaysia	3
15	*H. elephas* van Benthem Jutting, 1950	Bukit Tenggek, Pahang, Malaysia	3
16	*H. emergens* (van Benthem Jutting, 1950)	Bukit Chuping, Perlis, Malaysia	3, 7, 28
17	*H. frequens* (van Benthem Jutting, 1950)	Kota Tongkat, Pahang and Gunong Batu Kurau, Perak, Malaysia	3
18	*H. luctans* (van Benthem Jutting, 1950)	Gunong Pondok, Padang Rengas, Perak, Malaysia	3, 7, 32
19	*H. modestum* (van Benthem Jutting, 1950)	Gua Musang, Kelantan and Peninsular Siam, Malaysia	3, 7, 32
20	*H. serpa* (van Benthem Jutting, 1950)	Baling, Kedah, Malaysia; Tarutoa National Park, Satun and Krabi, Thailand	3
21	*H. troglodytes* (van Benthem Jutting, 1950)	Gua Bama, Padang Tengku, Pahang, Malaysia	3, 7, 28
22	*H. venustum* (van Benthem Jutting, 1950)	Gunong Pondok, Padang Rengas, Perak, and Kota Tongkat, Pahang, Malaysia	3
23	*H. saxicola* (van Benthem Jutting, 1960)	Kampong Tebing Tinggi, Kangar, Perlis, Malaysia	3, 31
24	*H. salpinx* (van Benthem Jutting, 1961)	Bukit Serdam, Pahang, Malaysia	3
25	*H. cambodjense* van Benthem Jutting, 1962	Kampot, Cambodia; Kien Giang, Vietnam	3
26	*H. dilatatum* van Benthem Jutting, 1962	Binh An Commune, Ba Nui Village, Mo So Cave, Kien Luong District Kien Giang, Vietnam	3
27	*H. rupestre* van Benthem Jutting, 1962	Kien Giang, Vietnam; Kampot, Cambodia	3
28	*H. torticollis* (van Benthem Jutting, 1962)	Phum Troung Mean, Banan District, Battambang, Cambodia	3, 23
29	*H. australe napieranum* (Solem, 1981)	Yammera Gap and Napier Range, Kimberley Region, Western Australia, Australia	3, 22
30	*H. paini* (F.G. Thompson & Dance, 1983)	Deer Cave, Melinau Paku Valley, Fourth Division, Sarawak, Borneo, Malaysia	3
31	*H. procerum* (F.G. Thompson & Dance, 1983)	Gunong Budah, Medalam Valley, Fifth Division, Sarawak, Borneo, Malaysia	3
32	*H. holimanae* F.G. Thompson & H.G. Lee, 1988	Kanchanaburi Agricultural College, Mueang District, Kanchanaburi, Thailand	3
33	*H. adela* (F.G. Thompson & Upatham, 1997)	Ban Na San District, Surat Thani, Thailand	17, 25
34	*H. burchi* (Panha, 1998)	Phatthalung, Surat Thani, and Phetchaburi, Thailand	3, 11, 17
35	*H. chedi* Panha, 1998	Tebpratan Nature Reserve area, Kamphaeng Phet, Thailand	3
36	*H. cucumense* Panha, 1998	Kangkrajan National Park, Phetchaburi, Thailand	3
37	*H. khaowongense* Panha, 1998	Takli District, Nakhon Sawan; Tepitak Tamaram Temple, Tepitak Mountain, Mueang Saraburi District, Saraburi; Muangon Cave, San Kam Pang District, Chiang Mai, Thailand	3, 13, 17
38	*H. edentatum* (Panha & J.B. Burch, 2002)	Tamphatai National Park, Ngao District, Lampang; Ban Pa Ngae Limestone Knoll, Padaet District, Chiang Rai, Thailand	3
39	*H. khaochongpran* (Panha & J.B. Burch, 2002)	Khaochongpran, Tao Pun Sub-District, Photharam District, Ratchaburi, Thailand	3
40	*H. muaklekense* (Panha & J.B. Burch, 2002)	Tepitak Mountain, Muaklek District, Saraburi, Thailand	3, 15, 17
41	*H. pendulum* (Panha & J.B. Burch, 2002)	Chonglom Mountain, Bhumiphol Dam Reservoir, Samngao District, Tak, Thailand	3
42	*H. phupaman* (Panha & J.B. Burch, 2002)	Phuphaman Mountain, Phetchabun, Thailand	3
43	*H. sichang* (Panha & J.B. Burch, 2002)	Sichang Island, Si Racha District, Chonburi, Thailand	3, 14, 17
44	*H. chatnareeae* (Panha & J.B. Burch, 2003)	Tam Sua Temple, Utong District, Suphanburi, Thailand	3
45	*H. diarmaidi* (Panha & J.B. Burch, 2003)	Pluangthong Mountain, Botong District, Chonburi, Thailand	2, 3, 17
46	*H. erawan* Panha & J.B. Burch, 2003	Erawan National Park, Sai Yok District, Kanchanaburi, Thailand	3
47	*H. panhai* J.B. Burch & Tongkerd, 2003	Chongkhaokad, Sai Yok District, Kanchanaburi, Thailand	3
48	*H. khaochakan* (Panha & J.B. Burch, 2003)	Chakan Mountain, Khaochakan Temple, Khaochakan District, Sa Kaeo, Thailand	2, 3, 17
49	*H. kohrin* (Panha & J.B. Burch, 2003)	Kohrin (Rin Island), Chonburi, Thailand	2, 3, 17
50	*H. surakiti* (Panha & J.B. Burch, 2003)	Puttabanpot Temple, Nawang District, Nong Bua Lamphu, Thailand	2, 3, 17
51	*H. taehwani* Panha & J.B. Burch, 2003	Central Area of Tamrong Temple, Mueang Phetchaburi District, Phetchaburi, Thailand	3
52	*H. khaowongkot* (Panha & J.B. Burch, 2004)	Khaowongkot, Ban Mi District, Lopburi, Thailand	3, 16, 17
53	*H. pattalungense* Panha & J.B. Burch, 2004	Limestone Hill in Ko Si Ko Ha (Ko Na Thewada), Phatthalung, Thailand	3
54	*H. smokon* (Panha & J.B. Burch, 2004)	Smokon Mountain, Ban Mi District, Lopburi, Thailand	3
55	*H. srakeoense* (Panha & J.B. Burch, 2004)	Plubpluengtong Limestone Hills, Sa Kaeo, Thailand	3, 23
56	*H. tridentatum* (Panha & J.B. Burch, 2004)	Plubpluengtong Limestone Hills, Sa Kaeo, Thailand	3, 16, 17
57	*H. utongensis* Panha & J.B. Burch, 2004	Tam Sua Hill, Utong District, Suphanburi, Thailand	3
58	*H. loei* Panha & Prateespasen, 2006	Limestone Hills in Loei, Phitsanulok, Khon Kaen and Nong Bua Lamphu, Thailand	3
59	*H. benetuitum* Vermeulen, Luu, Theary & Anker, 2019	Phnom Kampong Trach, Kampot, Cambodia	3
60	*H. chaunosalpinx* (Vermeulen, Luu, Theary & Anker, 2019)	Kampong Trach Area, Phnom Kampong Trach, Kampot, Cambodia	3
61	*H. depressum* (Vermeulen, Luu, Theary & Anker, 2019)	Phnom La’Ang Cave, Kampot Area, Kampot, Cambodia	3
62	*H. discobasis* Vermeulen, Luu, Theary & Anker, 2019	Phnom La’Ang, Kampot Area, Kampot, Cambodia	3
63	*H. cultura* (Tanmuangpak & Dumrongrojwattana, 2022)	Agricultural Areas in Mueang Loei District, Loei; Lampang and Chonburi, Thailand	3, 24
64	*H. khmerianum* (Sutcharit & Panha, 2023)	Phnom Sampeov Mountain, Banan District, Battambang, Cambodia	3, 23
65	*H. aunglini* (Tongkerd & Panha, 2024)	Kaw Gon Cave, Hpa-An, Kayin State, Myanmar	3, 27
66	*H. aenigma* Gojšina, Grego & Páll-Gergely, 2025	Pan Do Mi Mountain, Phon Tho Village, Kamarmaung, Kayin State, Myanmar	3
67	*H. aquila* Gojšina, Hunyadi & Páll-Gergely, 2025	Chap Phleung Mountain, Stung Treng Mekong Bridge, Steung Treng, Cambodia	3
68	*H. bubalus* Gojšina, Hunyadi & Páll-Gergely, 2025	Kodiang, Gua Kerbau, Kedah, Malaysia	3
69	*H. circumcarinatum* Gojšina, Auffenberg & Páll-Gergely, 2025	Taoist Buddhist Temple, Kanchanaburi, Thailand	3
70	*H. coriaceum* Gojšina & Páll-Gergely, 2025	Tanjung Rhu Beach, Langkawi Island, Malaysia	3
71	*H. fortunatum* Gojšina, Hunyadi & Páll-Gergely,2025	Khao Ok Thalu, Phattalung; & Songkhla, Thailand	3
72	*H. fungus* Gojšina, Hunyadi & Páll-Gergely, 2025	Stung Treng Mekong Bridge, Phnom Chhnok, Stung Treng , Cambodia	3
73	*H. geckophilum* Gojšina, Hunyadi & Páll-Gergely, 2025	Tham Khao Wongkot, Kaeng Hang Maeo District, Chanthaburi, Thailand	3
74	*H. iunior* Gojšina & Páll-Gergely, 2025	Ban Pha Tun, Tham Pha Tup Forest Park, Mueang Nan District, Nan, Thailand	3
75	*H. ophis* Gojšina, Hunyadi & Páll-Gergely, 2025	Khao Ok Thalu, Phatthalung, Thailand	3
76	*H. platybasis* Gojšina, Hunyadi & Páll-Gergely, 2025	Stung Treng Mekong Bridge, Chap Phleung Mountain, Steung Treng, Cambodia	3
77	*H. populare* Gojšina, Hunyadi & Páll-Gergely, 2025	Krabi, Chumphon and Ranong, Thailand	3
78	*H. sculpturatum* Gojšina, Hunyadi & Páll-Gergely, 2025	Stung Treng Mekong Bridge, Phnom Chhnok, Steung Treng, Cambodia	3
79	*H. similare* Gojšina, Hunyadi & Páll-Gergely, 2025	Wat Tham Khao Prathun, Khao Chamao District, Rayong, Thailand	3
80	*H. sorormajor* Gojšina, Hunyadi & Páll-Gergely, 2025	Stung Treng Mekong Bridge, Chap Phleung Mountain, Steung Treng, Cambodia	3
81	*H. sororminor* Gojšina, Hunyadi & Páll-Gergely, 2025	Stung Treng Mekong Bridge, Chap Phleung Mountain, Steung Treng, Cambodia	3
82	*H. tertiusfrater* Gojšina & Páll-Gergely, 2025	Gunong Subis; Sarawak & Sabah in Borneo, Malaysia	3
83	*H. torta* Gojšina, Auffenberg & Páll-Gergely, 2025	Nakhon Sawan, Thailand	3
84	*H. vesovici* Gojšina & Páll-Gergely, 2025	Gua Telinga, Kuala Tahan, Taman Negara, Jerantut, Pahang, Malaysia	3
85	*H. vicinum* Gojšina, Auffenberg & Páll-Gergely, 2025	Surat Thani, Thailand	3
86	*H. vujici* Gojšina & Páll-Gergely, 2025	Ban Non San Village, Nakhon Sawan, Thailand	3
87	*H. pongrati* sp. nov.	Si Bun Rueang District, Nong Bua Lamphu, Thailand	This study
88	*H. sichomphuense* sp. nov.	Si Chomphu District, Khon Kaen, Thailand	This study

References: 1 = Blanford (1863); 2 = Burch et al. (2003); 3 = Gojšina et al. (2025); 4 = Gude (1914); 5 = Inkhavilay et al. (2016); 6 = Inkhavilay et al. (2019); 7 = Maassen (2001); 8 = Möllendorff (1891); 11 = Panha (1998a); 12 = Panha (1998b); 13 = Panha (1998c); 14 = Panha and Burch (2002a); 15 = Panha and Burch (2002b); 16 = Panha et al. (2004); 17 = Panha and Burch (2008); 18 = Pilsbry (1917a); 19 = Pilsbry (1917b); 21 = Smith (1896); 22 = Solem (1981); 23 = Sutcharit et al. (2023); 24 = Tanmuangpak and Dumrongrojwattana (2022); 25 = Thompson and Upatham (1997); 26 = Tongkerd et al. (2013); 27 = Tongkerd et al. (2024); 28 = van Benthem Jutting (1949); 29 = van Benthem Jutting (1950); 30 = van Benthem Jutting (1952); 31 = van Benthem Jutting (1960); 32 = van Benthem Jutting (1962); 33 = Vermeulen and Whitten (1998); 34 = Vermeulen et al. (2019).

The two new species of *Hypselostoma* described in this paper were recently discovered on a wall in limestone hills covered with mixed-deciduous forest in Nong Bua Lamphu and Khon Kaen Provinces, northeastern Thailand. The external shell morphology of the two new species is very similar to that of *Hypselostoma phupaman* (Panha & J.B. Burch, 2002c) from Chaiyaphum Province (Panha and Burch 2008), with which they share the double-keeled last whorl, but from which they differ by their number of apertural barriers. These three species are also similar to *Hypselostoma torta* Gojšina, Auffenberg & Páll-Gergely, 2025 from Nakhon Sawan Province (Gojšina et al. 2025) with which they share their shell shape, as well as the strongly shouldered penultimate and last whorls. However, *H. torta* differs as it has only one keel on the last whorl, instead of two.

## ﻿Materials and methods

**Figure 2.**
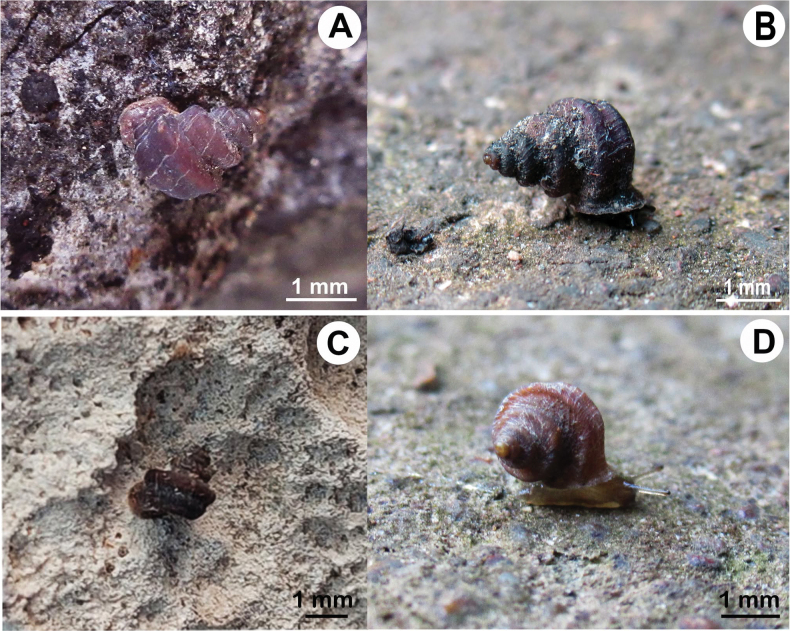
Living *Hypselostoma* spp. **A, B.***Hypselostoma pongrati* sp. nov. paratype NHLRU015; **C, D**. *Hypselostoma sichomphuense* sp. nov. paratype NHLRU033.

Microsnail specimens were collected during November–December 2021 and January 2022 from limestone hill in Nong Bua Lampu Province (Figs 1, 2A, B), and Khon Kaen Province (Figs 1, 2C, D), northeastern Thailand. Complete adult shells were used to count the number of whorls and measure shell height (**SH**), shell width (**SW**), aperture height (**AH**), and aperture width (**AW**) with digital vernier calipers (Electronic Digital Calliper S.H.). Shells and radulae were photographed by a scanning electron microscope (LEO 1450 VP) at the Microscope Center, Faculty of Science, Burapha University and Centre for Scientific and Technological Equipment at Suranaree University of Technology. Adult microsnails were dissected under a stereomicroscope to examine their genital system and extract their radula from the buccal mass. The radula sac was soaked in 5% NaOH solution to separate and clear the radula. Species identification was based on Pilsbry (1916–1918), van Benthem Jutting (1950), Panha and Burch (2008), and Gojšina et al. (2025). Shell characters were examined following Panha and Burch (2008) and Gojšina et al. (2025), whereas characters of genitalia and radula were studied following Dumrongrojwattana and Tanmuangpak (2020) and Tanmuangpak and Dumrongrojwattana (2022).

The type material was deposited in the following institutions: **NHLRU** Natural History Museum of Loei Rajabhat University (Loei, Thailand) and **NHMSU** Natural History Museum, Mahasarakham University (Maha Sarakham, Thailand).

## ﻿Results

### ﻿Taxonomy

**Family Hypselostomatidae Zilch, 1959**

#### ﻿Genus Hypselostoma

Taxon classificationAnimaliaEupulmonataHypselostomatidae

W.H. Benson, 1856

86B5930D-29B4-5365-B27A-C01885FAD0ED

##### Diagnosis.

Shell tiny, turbinate, high-spired, or discoidal. Peristome adnate or separated from the adjacent body whorl forming a tuba; edge of peristome expanded. Aperture with four or more parietal and angular apertural lamellae or plicae (barriers), except in *Hypselostoma edentatum* (Panha & J.B. Burch, 2002d) and *H. pongrati* sp. nov. which have no apertural barriers and *H. sichomphuense* sp. nov. which has only three barriers (Panha and Burch 2008; Gojšina et al. 2025). The genitalia of *Hypselostoma* possess a penis that is more swollen than the epiphallus; gametolytic sac connects to vagina very close to atrium, divided into two parts, with the proximal part swollen and larger than the penis (except in *H. cultura* (Tanmuangpak & Dumrongrojwattana, 2022), in which the gametolytic sac has a long duct between proximal and distal part, while there is no swollen proximal part in *H. depressispira* (Berry, 1963)). These anatomical characters differ from the genus *Aulacospira* Möllendorff, 1890, whose gametolytic sac connects to the vagina far from the atrium (Dumrongrojwattana and Tanmuangpak 2020)

#### ﻿ Hypselostoma pongrati

Taxon classificationAnimaliaEupulmonataHypselostomatidae

Tanmuangpak & S. Tumpeesuwan sp. nov.

095F7E0C-6B70-5DEC-8E9E-F734D112E162

https://zoobank.org/C545C51C-2464-468F-983B-9713B28FEFBC

**Figure 3.**
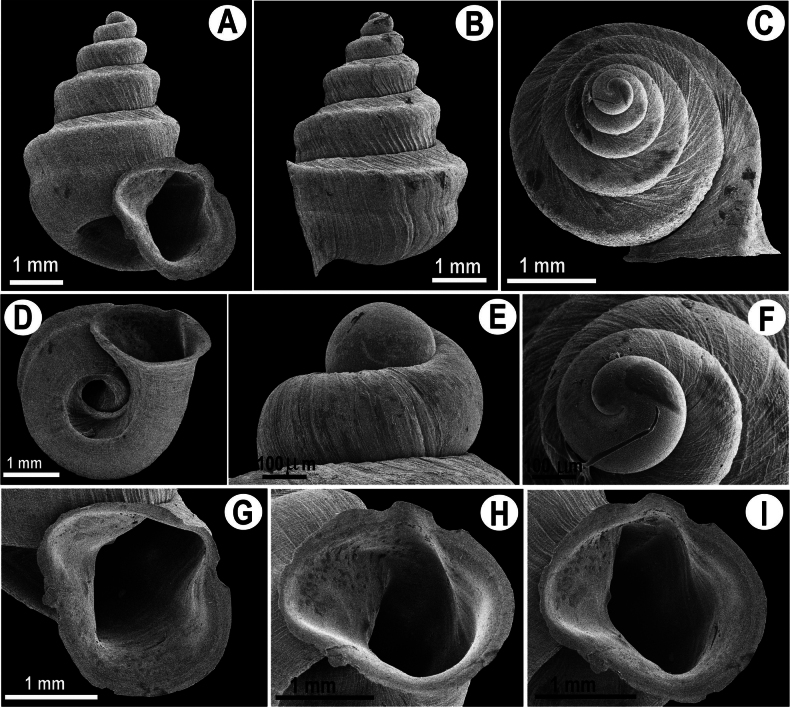
Shell morphology of *Hypselostoma pongrati* sp. nov. holotype NHLRU014. **A.** Apertural view; **B.** Lateral view; **C, E, F.** Protoconch view; **D.** Umbilical view apertural detail; **G–I.** Apertural view in different angles.

Figs 2A, B, 3A–I, 5A–E, 6A, B; Table 2

Hypselostoma sp. – Tanmuangpak & Kaewsawang, 2025: 20, 23, 25, 27, 29–31, fig. 2q.

##### Material examined.

***Holotype***: Thailand • 1 empty shell; Nong Bua Lamphu Province, Sri Bun Rueang District, Pha Sam Yod limestone hill (17°10'02.020"N, 102°02'03.020"E) covered by mixed-deciduous forest; 20.xi.2021, leg. Tanmuangpak, K. (NHLRU014) (Fig. 3A–E). ***Paratypes***: Thailand • 12 specimens preserved in 70% ethanol (NHLRU015–026); • five shells (NHLRU027–031); • three specimens preserved in 70% ethanol (NHMSU–00063–B); • two shells (NHMSU–00063). All paratypes collected from the same location as holotype, leg. Tanmuangpak, K. 10.xii.2021.

**Table 2.** Comparison of shell, radula, and genital system of *Hypselostoma pongrati* sp. nov., *H. sichomphuense* sp. nov., *H. cultura* and *Aulacospira vanwalleghemi*.

Characters	Species			
	*H. pongrati* sp. nov.	*H. sichomphuense* sp. nov.	*H. cultura*	*A. vanwalleghemi*
Shell:				
Shape	Conical	Concave-conical	Concave-conical	Conical-ovoid
Shell height (mm)	4.48–6.02	3.82–4.29	2.11–2.25	1.87–3.17
Shell width (mm)	3.07–4.12	3.12–4.12	2.50–2.80	1.74–3.28
Appearance of the last whorls	double keeled	double keeled	keeled at the center of the periphery	keeled above the center of the periphery
Apertural barrier	absent	3	26	absent
Radula Formula	(7-8):5:1:5:(7-8)	(9-10):7:1:7:(9-10)	8:5:1:5:8	(7-8):4:1:4:(7-8)
Genital system:				
Penial retractor muscle (pr)	absent	short tube	Bulged and curved	Short and rounded
Gametolytic sac	anterior portion bulge, larger than penis	anterior portion bulge, equal to penis	anterior portion bulge smaller than penis	anterior portion bulge, smaller than penis
	distal end long cylindrical and curved.	distal end long cylindrical and curved	distal end bulged	posterior portion long slender, curved
References	This study	This study	Tanmuangpak and Dumrongrojwattana (2022)	Dumrongrojwattana and Tanmuangpak (2020)

##### Measurements.

Holotype: SH = 4.48 mm, SW = 3.34 mm, AH = 1.95 mm, AW = 1.92 mm. Paratypes (19 specimens measured): SH = 4.48–6.02 mm (5.58 ± 0.42 mm), SW = 3.07–4.12 mm (3.72 ± 0.36 mm), AH = 1.95–2.64 mm (2.24 ± 0.20 mm), AW = 1.68–2.42 mm (1.99 ± 0.16 mm) (Fig. 7).

##### Diagnosis.

This new species has a prominent keel on the upper part of the last whorl and a weaker keel on the lower part. Apertural barriers absent (Fig. 3A, G, H, L). Terminal part of the last whorl is adnated to penultimate whorl.

##### Description.

***Shell*** (Fig. 3A–I) concave-conical, high spired, with 4¾ whorls. Large size for the genus, with shell height 4.48–6.02 mm, shell width 3.07–4.12 mm. The suture is deep. Protoconch smooth, with 1–1½ whorls that gradually increase in size to the teleoconch whorls. Teleoconch with 3–3¾ whorls, very fine spiral striation the body whorl widest, possesses two keels (upper one more prominent than the lower one). Apertural barriers absent. Peristome thickened and expanded. The parietal and convex angulo-palatal embayments form a sinulus, thickened, and expanded. The basal peristome is smooth and expanded, columellar side is more expand than the angulo-palatal side.

***Genital system*** (Fig. 6A, B). Atrium shorter than the vagina. Penis shorter than the epiphallus, its anterior portion is a short and bulged tube, and the distal end of the penis bulge. Epiphallus connects to the distal end of the penis, shorter than vas deferens, anterior portion slender and cylindrical, its central portion more slender than the anterior portion, posterior portion curved, white glossy. Penial retractor muscle absent. Vas deferens long, slender, entering the epiphallus apically. The vagina and free oviduct are cylindrical, and the vagina is shorter than the free oviduct. The gametolytic sac is a long tube, with a swollen proximal part, and larger than penis, surrounded by a thin sheath and connected to the proximal part of the vagina, whose distal end is long, slender, and curved. Uterus large, with very thin prostate gland attached to it. Hermaphroditic duct loosely convoluted. The albumen gland large and yellowish. Dart apparatus absent.

***Radula*** (*n* = 4) (Fig. 5A–E). Radula comprises 173–184 (179 ± 4.93) transverse rows of teeth each row containing 25–27 teeth. Radula formula (7-8)+5+1+5+(7-8). Central tooth small, unicuspid, elongated triangular. Lateral teeth bicuspid and asymmetrical, consisting of a large lanceolate endocone and smaller elongated triangular ectocone. Five lateral teeth on each side of the central tooth, the first tooth largest, and gradually smaller outwards. Marginal teeth start at 6^th^ tooth outwards from central teeth, which almost changed to tricuspids. On each outer side of the lateral teeth there are seven or eight marginal teeth.

##### Etymology.

This new species is dedicated to the late Pongrat Dumrongrojwattana, our highly respected senior who was an expert on microsnails and who provided us with many land snails references, knowledge, and inspiration.

##### Animal and ecology.

Living animals have a cream-colored body and foot, while their head and tentacles are rather black, with black eyes located at the tip of the ocular tentacle. This species was found on the limestone wall in the mixed-deciduous forest (Fig. 2A, B).

##### Distribution.

This species was found only on limestone wall in Pha Sam Yod, Si Bun Rueang District, Nong Bua Lamphu Province.

##### Remarks.

*Hypselostoma pongrati* sp. nov. from Si Bun Rueng District, Nong Bua Lam Phu Province, is quite different in shell shape from almost all other *Hypselostoma* species in Thailand. It is similar in shell shape to the geographically close *H. phupaman* from Chaiyaphum Province and *H. sichomphuense* sp. nov. from Si Chomphu, Khon Kaen Province. The unique characteristics of *H. pongrati* sp. nov. are the lack of apertural barriers and the last whorl adnate to the penultimate whorl, peristome thick, expanded and not reflected. It is strongly expanded at the parietal side where it leans against penultimate whorl and forms a thick crescent callus. Three species possess a double-keeled last whorl, but the lower keel is weak in both *H. pongrati* sp. nov. and *H. sichomphuense* sp. nov. Other similar species are *H. torta* from Nakhon Sawan Province, Thailand and *H. platybasis* from Steung Treng Province, Cambodia (Gojšina et al. 2025). In *H. torta* and *H. pongrati* sp. nov. all teleoconch whorls are shouldered, but in *H. torta* the last whorl is detached from the penultimate whorl. There are four weak apertural barriers. The columellar barriers form as a rather strong swelled part of the aperture which forms a prominent basal furrow below it. *Hypselostoma platybasis* differs from *H. pongrati* sp. nov. by possesses bluntly keeled penultimate at the center of the periphery and shouldered last whorl. Last whorl very slightly detached from the penultimate whorl and with a sharp shoulder. Apertural barriers few and relatively weak. In *H. pongrati* sp. nov. there is no penial retractor muscle, and the free oviduct is longer than the anterior portion of the gametolytic sac. Conversely, in *H. sichomphuense* sp. nov. there is a penial retractor muscle, and the free oviduct is shorter than the anterior portion of the gametolytic sac.

#### ﻿ Hypselostoma sichomphuense

Taxon classificationAnimaliaEupulmonataHypselostomatidae

Tanmuangpak & S. Tumpeesuwan sp. nov.

2F106A50-5E7D-525E-8B8B-6631EFACD4B5

https://zoobank.org/EAE8D792-E347-4F2C-9BDB-2291A398D89C

**Figure 4.**
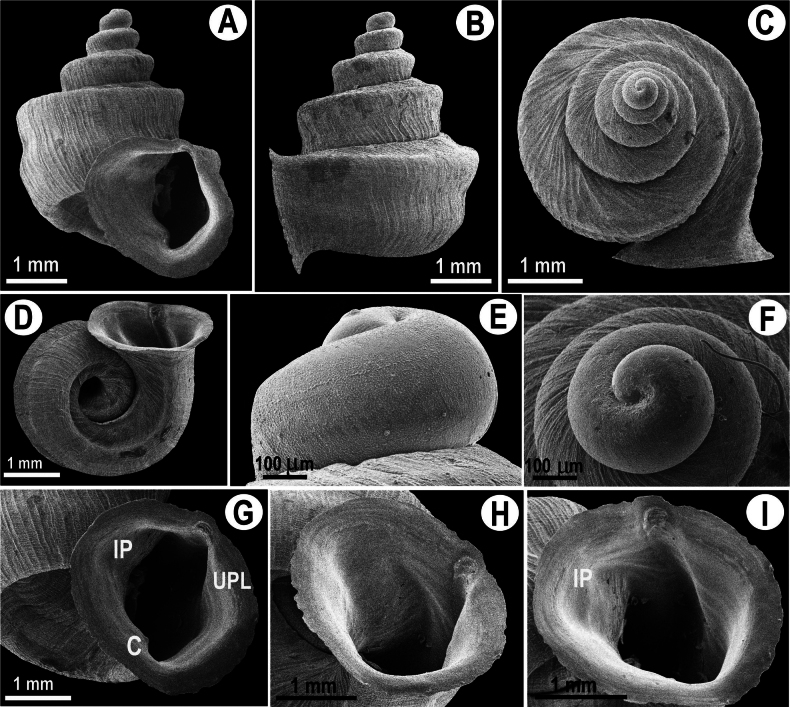
Shell morphology of *Hypselostoma sichomphuense* sp. nov. holotype NHLRU032. **A, G–I.** Apertural view; **B.** Lateral view; **C, E, F.** Protoconch view; **D.** Umbilical view; **A, G–I.** Apertural view in different angles. Abbreviations for apertural teeth: UPL = upper palatal plica; C = columellar fold; IP = infraparietal plica.

**Figure 5.**
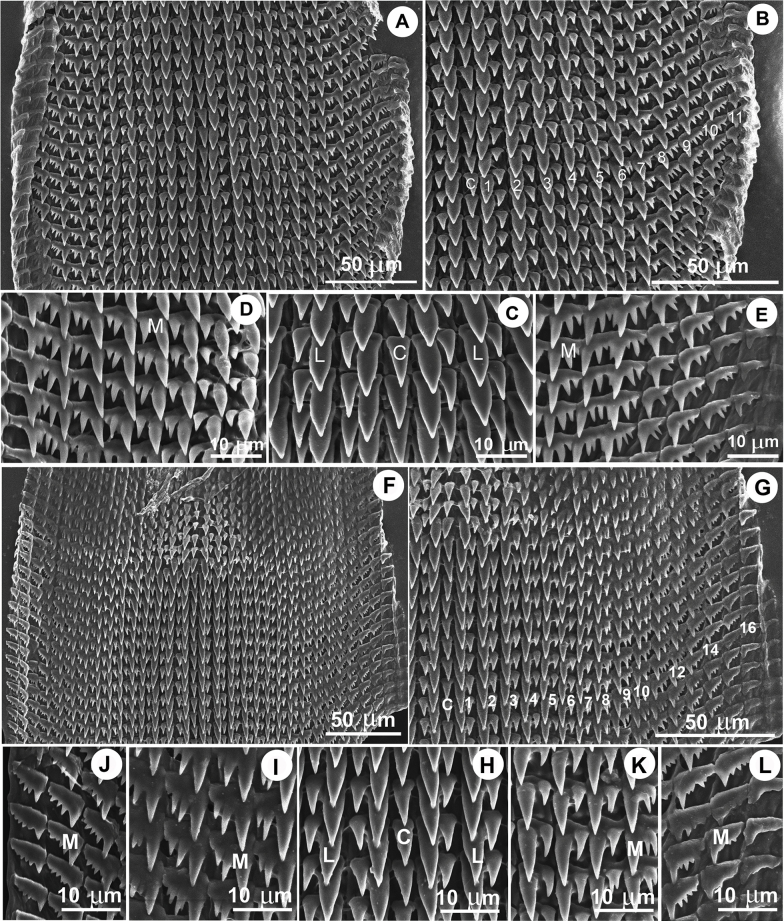
Radula morphology of *Hypselostoma* spp. **A–E.***Hypselostoma pongrati* sp. nov. paratype NHLRU015; **F–L.***Hypselostoma sichomphuense* sp. nov. paratype NHLRU034. Abbreviations: C = central tooth; L = lateral teeth; M = marginal teeth.

**Figure 6.**
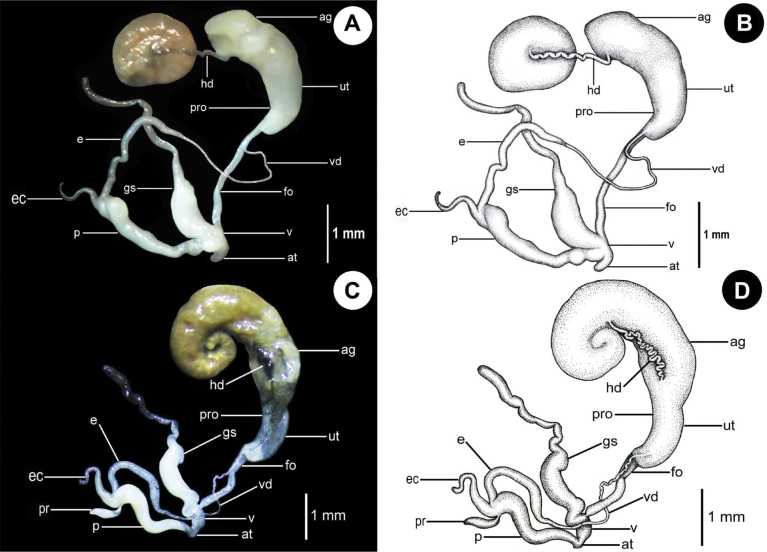
Genital system of *Hypselostoma* spp. **A, B.***Hypselostoma pongrati* sp. nov., paratype (NHLRU015); **A.** Genital system; **B.** Schematic drawing of the genital system; **C, D.***Hypselostoma sichomphuense* sp. nov., paratype (NHLRU034); **C.** Genital system; **D.** Schematic drawing of the genital system. Abbreviations: ag = albumen gland; at = atrium; e = epiphallus; ec = epiphallic caecum; fo = free oviduct; hd = hermaphroditic duct; p = penis; pr = penial retractor muscle; pro = prostate; gs = gametolytic sac; ut = uterus; v = vagina; vd = vas deferens.

Figs 2C, D, 4A–I, 5F–L, 6C, D; Table 2

##### Material examined.

***Holotype***: Thailand • 1 empty shell Khon Kaen Province, Si Chomphu District, limestone wall in mixed-deciduous forest, Phu Pha Kham (16°50'22.001"N, 102°03'59.001"E), 21.xi.2021, leg. Tanmuangpak, K. (NHLRU032) (Fig. 3G–K). ***Paratypes***: Thailand • 12 specimens preserved in 70% ethanol (NHLRU033–044) (Fig. 2C, D); • two shells (NHLRU045–046); • four specimens preserved in 70% ethanol (NHMSU–00064-B); • one shell (NHMSU–00064). All paratypes collected from the same location as holotype, leg. Tanmuangpak, K. 11.xii.2021.

##### Measurements.

**Figure 7.**
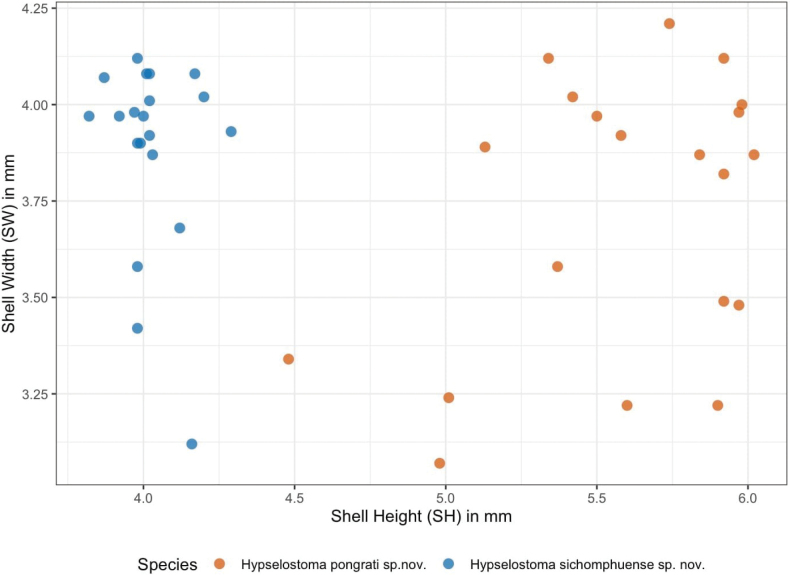
Shell dimension of *Hypselostoma pongrati* sp. nov. and *Hypselostoma sichomphuense* sp. nov.

Holotype: SH = 4.20 mm, SW = 4.02 mm, AH = 2.14 mm, AW = 2.24 mm. Paratypes: (19 specimens measured). SH = 3.82–4.29 mm (4.03 ± 0.11 mm), SW = 3.12–4.12 mm (3.88 ± 0.25 mm), AH = 1.84–2.27 mm (2.02 ± 0.12 mm), AW = 1.67–2.24 mm (2.01 ± 0.13 mm) (Fig. 7).

##### Diagnosis.

This new species differs from *Hypselostoma phupaman* by the presence of a non-prominent lower keel on the last whorl and the absence of basal and parietal apertural barriers. Aperture has three smooth tooth-like swellings (Fig. 4A, G–I), whereas there are no apertural barriers in *H. pongrati* sp. nov. (Fig. 3A, G–I). Spire shorter than the spire of *H. pongrati* sp. nov.

##### Description.

***Shell*** (Fig. 4A–I) concave-conical, wide-spired, with 4½ whorls. Large size for the genus, with shell height 3.82–4.29 mm, shell width 3.12–4.12 mm. The suture is deep. Protoconch pitted, without spiral pattern, with one whorl gradually increasing in size to the teleoconch whorls. The teleoconch has 3½ whorls, a very fine spiral surface, with growth lines. The body whorl widest, double keeled (prominent upper and weak lower keel). The peristome is thickened and expanded. Aperture has three smooth, tooth-like swellings, viz. an upper palatal plica, a columellar fold, and a infraparietal plica.

***Genital system*** (Fig. 6C, D). Atrium is shorter than the vagina. Penis is longer than the epiphallus, its anterior portion is a short and gradually bulged tube, and the distal end of its posterior portion is bulged as well. Epiphallus is connected to the distal end of the penis. Epiphallus shorter than vas deferens, its anterior portion slender and cylindrical, its central portion gradually bulges, and its posterior portion is a slender tube, white and glossy. Epiphallic caecum is a short tube, attached to the anterior portion of epiphallus. The penial retractor muscle is shorter than the epiphallus, attached to the distal end of the penis. Vas deferens long, slender, entering epiphallus apically. The vagina and free oviduct are cylindrical, and the vagina is shorter than the free oviduct. The gametolytic sac is a long tube, its bulging anterior portion is surrounded by a thin sheath and connected to vagina, the distal end is a long slender and curved tube. The uterus is large, with a very thin prostate gland attached to it. The hermaphroditic duct is loosely convoluted. The albumen gland is yellowish and large. Dart apparatus absent.

***Radula*** (*n* = 3) (Fig. 5F–L). Radula comprises of 154–165 (159 ± 5.56) rows of teeth, each row with 33–35 teeth. Radula formula: (9–10) +7+1+7+ (9–10). Central tooth small, unicuspid, and triangular. Lateral teeth bicuspid and asymmetrical, consisting of a large endocone and a smaller ectocone. Seven lateral teeth on each side of the central tooth; the first tooth is largest, and the other teeth are sequentially smaller. Marginal teeth are irregular, unequally tricuspid, with an endocone larger than the ectocone and gradually change to polycuspid outwards (Fig. 5I–L). There are nine or ten marginal teeth on each outer side of the lateral teeth (Fig. 5F–L).

##### Etymology.

The specific epithet *sichomphuense* refers to Si Chomphu District, Khon Kaen Province, northeastern Thailand, where this species was discovered.

##### Animal and ecology.

Living animals have cream-colored bodies, with pale brown head and tentacles and black eyes at the tip of the ocular tentacle. This species was found on the limestone wall covered by mixed-deciduous forest (Fig. 2C, D).

##### Distribution.

Limestone wall at Phu Kham, Si Chomphu District, Khon Kaen Province.

##### Remarks.

*Hypselostoma sichomphuense* sp. nov. is quite different in shell shape from all other *Hypselostoma* species in Thailand, except for the geographically close *H. pongrati* sp. nov. The unique characteristics of *H. sichomphuense* sp. nov. are the three tooth-like swellings in the aperture and the last whorl adnate to the penultimate whorl.

## ﻿Discussion

The general shape of the radula teeth of the two new species is very similar to that of *Hypselostoma cultura*, but their formulae are different: (7–10):(5–7):1:(5–7):(7–10) in the new species versus 8:5:1:5:8 in *H. cultura* (Tanmuangpak and Dumrongrojwattana 2022).

Compared with the genital systems of *H. cultura* (see Tanmuangpak and Dumrongrojwattana 2022) and *Aulacospira* spp. (see Dumrongrojwattana and Tanmuangpak 2020), these two new species have a large cylindrically shaped penis, which is relatively long and whose distal end bulges. The epiphallus is more elongated with a cylindrical shape but shorter than the penis. The gametolytic sac is very long, the anterior to central portion bulged, equal to larger than penis and connected to the vagina, and the central to posterior portion is slenderer and long, with the distal end curved.

Phylogenetic analysis of DNA sequence data of some Asian land microsnails showed the incongruence between shell-based taxonomy and DNA-based phylogenetic relationships (Chiba 1999; Tongkerd et al. 2004a; Hirano et al. 2014). For example, DNA-based phylogenic analysis of Thai microsnails revealed that *Gyliotrachela*, *Hypselostoma* and *Anauchen* are paraphyletic, e.g., *Aulacospira smaesarnensis* Panha & Burch, 2001 nested within *Gyliotrachela* (Tongkerd et al. 2004a). In this phylogenetic tree, the leaf litter inhabiting *H. panhai*, with its last whorl adnated to the penultimate whorl, whereas the limestone-dwelling *H. erawan* as sister group has the last part of last whorl detached from the penultimate whorl. Tongkerd et al. (2004a) suggested that the loss of this ancestral shell character state may have been driven by selective pressures in the new leaf litter habitat. These results suggest that the use of apertural dentition is not suitable for diagnostic generic characters because of ecological transitions can lead to morphological change. In this study, the species with a double-keeled last whorl seem to have fewer apertural barriers, ranging from six in *H. phupaman* (Panha & Burch, 2008) to none in *H. pongrati* sp. nov. The reduction of apertural barriers may be due to decrease in predation pressure by specific predators (Páll-Gergely et al. 2022), which is an interesting point for further research.

In the period 2002–2023, many new species of land snails were described from the isolated limestone hills in Chaiyaphum-Khon Kaen-Nong Bua Lamphu-Loei Areas, for example, *Hypselostoma phupaman* (Panha & J.B. Burch, 2002); *H. surakiti* (Panha & J.B. Burch, 2003); *H. loei* Panha & Prateespasen, 2005; *Phuphania costata* C. Tumpeesuwan & S. Tumpeesuwan, 2014; *Sinoennea loeiensis* Tanmuangpak & S. Tumpeesuwan, 2015 in Tanmuangpak et al. (2015); *Sesara triodon* Tanmuangpak & Tumpeesuwan, 2016 in Tanmuangpak et al. (2016); *Aenigmatoconcha clivicola* C. Tumpeesuwan & S. Tumpeesuwan, 2017; *Landouria strobiloides* C. Tumpeesuwan & S. Tumpeesuwan, 2019; *Landouria circinata*Nahok et al., 2021; *L. tuberculata*Nahok et al., 2021; *L. trochomorphoides*Nahok et al., 2021; *L. chloritoides*Nahok et al., 2021; and *L. elegans*Nahok et al., 2021; *H. cultura* Tanmuangpak & Dumrongrojwattana, 2022; and *Rhiostoma ebenozostera* Tongkerd & Panha, 2023 in Tongkerd et al. (2023). These new species described from this limestone area confirm that this area is very important for biodiversity conservation and needs continuous studies and protection.

## Supplementary Material

XML Treatment for Hypselostoma

XML Treatment for Hypselostoma pongrati

XML Treatment for Hypselostoma sichomphuense

## ﻿Additional information

### Use of AI

No use of AI was reported.

### Author ORCIDs

Kitti Tanmuangpak https://orcid.org/0000-0003-2968-3506

Benchawan Nahok https://orcid.org/0009-0009-1788-6788

Utain Chanlabut https://orcid.org/0000-0001-8275-7434

Chanidaporn Tumpeesuwan https://orcid.org/0000-0003-2066-1271

Sakboworn Tumpeesuwan https://orcid.org/0000-0001-5900-7862

### Data availability

All of the data that support the findings of this study are available in the main text.
